# Autologous Doping with Cryopreserved Red Blood Cells – Effects on Physical Performance and Detection by Multivariate Statistics

**DOI:** 10.1371/journal.pone.0156157

**Published:** 2016-06-10

**Authors:** Christer B. Malm, Nelson S. Khoo, Irene Granlund, Emilia Lindstedt, Andreas Hult

**Affiliations:** 1 Sports Medicine Unit, Umeå University, Umeå, Sweden; 2 Winternet, Boden, Sweden; Université Claude Bernard Lyon 1, FRANCE

## Abstract

The discovery of erythropoietin (EPO) simplified blood doping in sports, but improved detection methods, for EPO has forced cheating athletes to return to blood transfusion. Autologous blood transfusion with cryopreserved red blood cells (RBCs) is the method of choice, because no valid method exists to accurately detect such event. In endurance sports, it can be estimated that elite athletes improve performance by up to 3% with blood doping, regardless of method. Valid detection methods for autologous blood doping is important to maintain credibility of athletic performances. Recreational male (N = 27) and female (N = 11) athletes served as Transfusion (N = 28) and Control (N = 10) subjects in two different transfusion settings. Hematological variables and physical performance were measured before donation of 450 or 900 mL whole blood, and until four weeks after re-infusion of the cryopreserved RBC fraction. Blood was analyzed for transferrin, iron, Hb, EVF, MCV, MCHC, reticulocytes, leucocytes and EPO. Repeated measures multivariate analysis of variance (MANOVA) and pattern recognition using Principal Component Analysis (PCA) and Orthogonal Projections of Latent Structures (OPLS) discriminant analysis (DA) investigated differences between Control and Transfusion groups over time. Significant increase in performance (15 ± 8%) and VO_2max_ (17 ± 10%) (mean ± SD) could be measured 48 h after RBC re-infusion, and remained increased for up to four weeks in some subjects. In total, 533 blood samples were included in the study (Clean = 220, Transfused = 313). In response to blood transfusion, the largest change in hematological variables occurred 48 h after blood donation, when Control and Transfused groups could be separated with OPLS-DA (R^2^ = 0.76/Q^2^ = 0.59). RBC re-infusion resulted in the best model (R^2^ = 0.40/Q^2^ = 0.10) at the first sampling point (48 h), predicting one false positive and one false negative. Over all, a 25% and 86% false positives ratio was achieved in two separate trials. In conclusions, autologous re-infusion of RBCs increased VO_2max_ and performance as hypothesized, but hematological profiling by multivariate statistics could not reach the WADA stipulated false positive ratio of <0.001% at any time point investigated. A majority of samples remained within limits of normal individual variation at all times.

## Introduction

In endurance sports, maximal oxygen uptake (VO_2max_) is an important factor for performance [1, 2]. Limitations in VO_2max_ are, assuming normal lung function and sea-level oxygen tension, maximal cardiac output (Q_max_) [1, 3], oxygen carrying capacity of the blood [4, 5] and total hemoglobin mass [6]. Extraction of available oxygen to working muscle is also a factor, at least in elite athletes [7]. Depending on mode and duration of work being performed, and the mode of testing, we have found the influence of VO_2max_ on physical performance to range from 62% to 88% in cross country skiing [2] and 42% to 79% in firefighting [8]. Others have previously reached similar models for running [9, 10], orienteering [11] cycling [12, 13], swimming [14, 15] and triathlon [16]. In intermittent exercise, such as soccer, the influence of VO_2max_ on performance is not known [17–19], possible due to difficulties in measuring the dependent outcome variable (performance) in team sports. Consequently, different methods to enhancing oxygen delivery can be used by cheating athletes, and the effects on physical performance can be substantial [20].

Initially, blood transfusion was used to enhance military aviation pilots’ work capacity to fly at high altitude during WWII, when pressurized cockpits were not used [21]. Later, submaximal [22] and maximal [23] running performance was shown to improve with blood transfusion. The discovery of erythropoietin (EPO) [24] simplified blood doping in sports, supplementing blood donation, storage and re-infusion. Similar performance enhancements of 6–12% could now be achieved by a simple recombinant human (rh) EPO injection [25–27]. In a review on blood doping published in 1989, Jones and Tunstall [28] describe increases in performance and VO_2max_ ranging between 0% and 40%, depending on the subjects included and methods used for both testing and doping. From the summarized literature, it can be estimated that elite athletes may improve performance by up to 3% with blood doping, regardless of method [29–31]. This enhancement is equivalent to, for example, seven minutes faster winning time in the 90 km cross country ski race Vasaloppet, 20–30 seconds faster time in any given 5000 m run at world class level, and four minutes faster finishing time in a marathon race. In cycling, a 3% increase in performance translate to a more than two hour faster winning time in Tour de France 2014.

The World Anti-Doping Agency (WADA) has banned the use many techniques to increase the oxygen carrying capacity of blood, including; blood transfusion, hormone injections, artificial oxygen carriers, allosteric Hb modulators and genetic manipulations [32].

While methods to detect rhEPO [33, 34] and homologous blood transfusion [35] have successfully been developed, no direct method is available for autologous blood transfusion [36, 37]. Currently the Athlete Biological Passport (ABP) [38, 39] is the best practice, although with known limitations [36, 40]. It can therefore be assumed that cheating athletes has returned to the practice of blood transfusions.

One study [41] investigated the sensitivity of doping detection based on measurements of hemoglobin, hematocrit, reticulocyte percentage (%ret) serum EPO and soluble transferrin receptor. The results showed a significant increase in %ret and decrease in [Hb] as a consequence of the donation. However, donation time is difficult to predict, since cryopreservation makes it possible to store blood for extended time (years to decades). Hematological [42] as well as performance enhancing effects may last for weeks to months in both men [5] and women [43]. Thus, sampling blood from any athlete with intention to detect a blood transfusion is subject to chance, when both donation and re-infusion can be done at any time out-of-Competition. An extensive review of various methods to detect autologous blood doping was recently published [44].

The present study aimed to investigate changes in physical performance, aerobic power and hematological variables that arise from autologous blood transfusion of cryopreserved RBCs. Also, if multivariate statistics can be used to predict subjects into Clean and Transfused groups at any of measured time points.

## Method

### Study design

**Fig 1:**
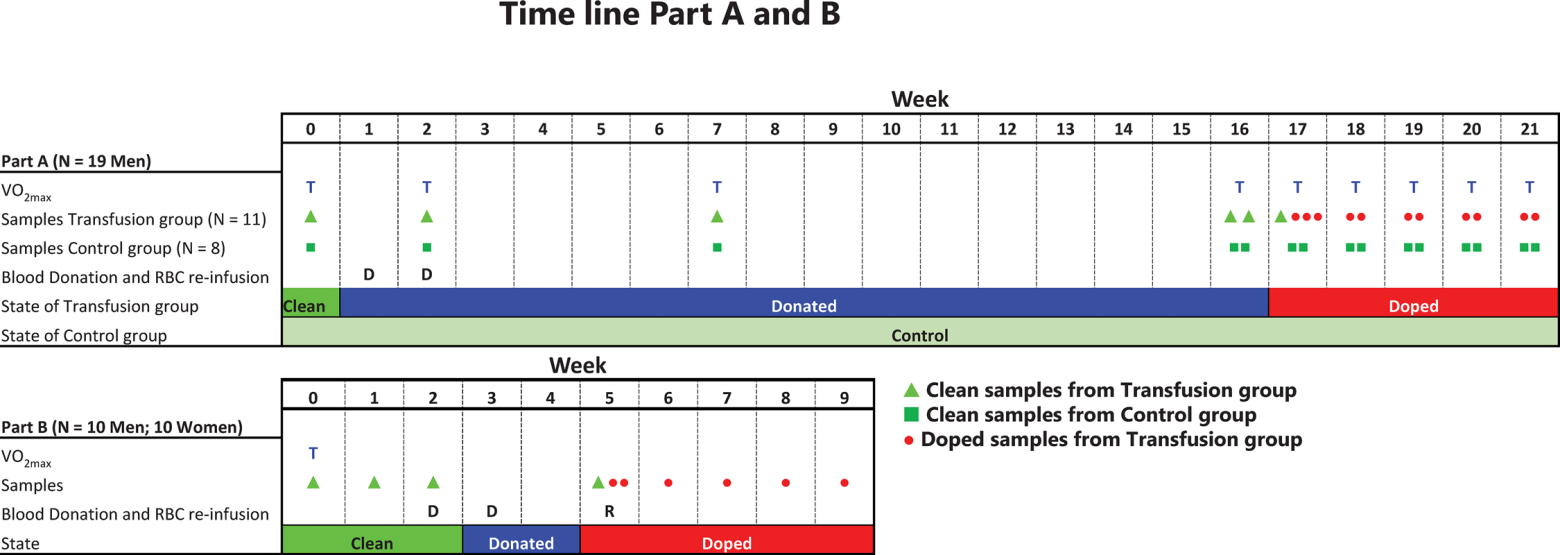
Time lines showing blood donation and re-infusion, blood sampling and VO_2max_ testing from Part A of the Study and B of the study. In total, 19 men and ten women have participated, and 430 blood samples were taken (257 in Part A of the Study and 173 in Part B of the Study). D; Donation of one unit (450 mL) blood (for women in Part B of the Study at Week 3 only). R; Re-infusion of RBC. T; Testing of physical performance. ▲Clean samples from the Transfusion group; ■ Clean samples from Control group; ●Samples after RBC re-infusion.

In total, 29 individuals participated in the study, randomized to Transfusion and Control groups in Part A of the Study, while in Part B of the Study subjects served as their own controls (Fig 1). Hematological variables and VO_2max_ (Part A of the Study only) were measured before donation, after donation and after re-infusion of RBC (Fig 1). In Part A of the Study, blood samples were taken at 17 times and nine treadmill running performance tests were completed. In Part B of the Study, ten blood samples were taken, and a tests for physical performance only executed as subject characterization prior to blood donation. Hematological variables measured are listed in Table 1.

**Table 1:** Hematological variables analyzed in blood samples.

Test	Explanation and unit
S-Transferrin	Serum Transferrin (g·L^-1^)
Hb	Hemoglobin in blood (g·L^-1^)
EPC	Erythrocyte Particle Concentration of red blood cells in the blood (n·10¹²·L^-1^)
EVF	Erythrocyte Volume Fraction (previously termed hematocrit)
MCV	Mean Corpuscular Volume of red blood cells (fL)
MCHC	Mean Corpuscular Hemoglobin Concentration (g·L^-1^)
EPO	Erythropoietin (IU/L), Part A of the Study only
Reticulocytes	n·10⁹·L^-1^, Part A of the Study only
Reticulocytes/RBC	Ratio between Reticulocytes and RBC (%), Part A of the Study only
Leukocytes	10^9^·L^-1^, Part B of the Study only
Neutrophils	10^9^·L^-1^, Part B of the Study only
Lymphocytes	10^9^·L^-1^, Part B of the Study only
Monocytes	10^9^·L^-1,^ Part B of the Study only
Eosinophils	10^9^·L^-1,^ Part B of the Study only
Basophils	10^9^·L^-1,^ Part B of the Study only
Thrombocytes	10^9^·L^-1,^ Part B of the Study only
Bilirubin	μmol·L^-1^, Part B of the Study only
Iron	μmol·L^-1^, Part B of the Study only
Transferrin	g·L^-1^, Part B of the Study only

### Subjects

Part A of the Study: Seventeen healthy males, recreational athletes, participated in the study. Anthropometric parameters and performance variables were not significantly different between the Control and Transfusion groups (Table 2). Part B of the Study: Eleven healthy women and ten healthy men were recruited for donation on one (women) and two (men) 450 mL units of blood. Two female subjects were excluded in the statistical analysis due to less than three data points after RBC re-infusion. Two men and six women participated in the transfusion experiment without completing the physical performance test (Table 3).

**Table 2:** Subject characteristics (Part A of the Study).

Variable	Control	Transfusion
N	7	10
Age (year)	32 ± 4	34 ± 8
Body mass (kg)	84.8 ± 11	83.1 ± 10
Resting systolic blood pressure (mmHg)	130 ± 11	132 ± 9
Resting diastolic blood pressure (mmHg)	65 ± 6	67 ± 8
VO₂_max_ (ml·min^-1^·kg^-1^)	56 ± 6	57 ± 5
VO_₂ max_ (ml·min^-1^)	4762 ± 535	4732 ± 499
Max blood lactat (mM)	12 ± 3	13 ± 2
Time to exhaustion (sec)	382 ± 46	417 ± 18

Mean ± SD.

**Table 3:** Subject characteristics (Part B of the Study).

Variable	Men	Women
N	10	11
Age (year)	28 ± 6	26 ± 3
Body mass (kg)	83 ± 6	63 ± 8
VO_₂ max_ (ml·min^-1^), N; Men = 8, Women = 4	4 ± 1	3 ± 0
VO₂_max_ (ml·min^-1^·kg^-1^) N; Men = 8, Women = 4	53 ± 8	48 ± 5
Max blood lactat (mM) N; Men = 8, Women = 4	13 ± 3	10 ± 3

Mean ± SD.

### Ethics

The Central Research Ethics Committee for Sweden approved the study on April 7, 2009 (Ö 1–2009), and the Research Ethics Committee for Northern Sweden at Umeå University approved additional amendments (2011-408-32M, 2013-218-32M): The study was conducted in accordance with the WMA Declaration of Helsinki–Ethical Principles for Medical Research Involving Human Subjects 2008. All participants provided written informed consent.

### Blood samples and transfusion

Part A of the Study: One week after baseline performance testing and blood sampling, 450 mL whole blood was donated, followed by a second 450 mL donation on Week 2, for a total of 900 mL blood. Blood donation followed standard hospital procedures in Sweden [45] regarding illnesses, staying abroad, tattoos or drugs. Donations were performed at Björknäs Health Care Center, Boden, Sweden. Blood was cryopreserved at Huddinge University Hospital, Huddinge, Sweden and re-infused at Sunderby Hospital, Luleå, Sweden. Red blood cells were isolated and processed for cryopreservation as described elsewhere [46] and RBCs stored at -80°C for 15 and 16 weeks, unit one and two, respectively, before RBC re-infusion. To restore iron supplies the Transfusion group received 100 mg iron supplementation per day during the entire study. Re-infusion of both units washed RBC took place 16 weeks after donation of the first unit. Blood samples (1 x 4.5 mL EDTA and 3 x 4.5mL serum) were taken as indicated in Fig 1. EDTA tubes were analyzed for standard hematology by the clinical laboratory at Sunderby Hospital, with remaining blood treated for a separate proteomic analysis of RBC (not reported in this paper). Serum was stored for EPO analysis (Access 2 chemiluminescent, Beckman Coulter, CA, USA) and for use in a separate study on cytokines [46]. Over a period of 21 weeks, a maximum of 235 mL venous blood in 17 samples was taken for analysis from each subject.

Part B of the Study: After an initial treadmill running test of VO_2max_, one blood sample was taken each week for three weeks to establish individual baseline values, similar to the ABP (Fig 1). On week 2 and 3, one unit (450 mL) whole blood was donated by all men, and all women donated 450 mL on week 3 only. Blood sampling, donation and re-infusion was all performed at the Center for apheresis and stem cell handling, Karolinska University Hospital, Huddinge, Sweden. All methods for blood sampling and preparation, including cryopreservation of RBCs were replicated form Part A of the Study, with the exception of EPO, which was not analyzed for. In addition, a white blood cell count was performed, and OFF-hr score was calculated as $\text{Hb}-60\sqrt{Ret\left(\%\right)}$ [47] in Part B of the Study only

### Aerobic capacity and physical performance

Aerobic work capacity was investigated by VO_2max_ (L·min^-1^) and physical performance as time to exhaustion (sec) during treadmill running. In Part A of the Study, performed at Winternet, Boden, Sweden, all subjects were tested the week before and 48 h, five and 15 weeks after donation, then at 48 h and at weeks 1, 2, 3 and 4 after RBC re-infusion. Jaeger Oxycon Pro metabolic unit was used and calibrated according to manufacturer’s instructions (Jaeger Oxycon Pro; Care Fusion, San Diego, USA, with Hans Rudolph accessories; Hans Rudolph Inc., Kansas city, USA) while running on a Rodby treadmill (Rodby Innovation AB, Sweden). In Part B of the Study, the same procedure was followed when tested at The Swedish School of Sport and Health Sciences, Stockholm, Sweden, but only once before the first blood sample.

Blood pressure was manually check at rest before each testing session (Part A of the Study), and heart rate monitored during running (Polar heart rate monitor S810; Polar Electro Oy, Kempele, Finland). Fingertip blood lactate was analyzed at rest and three minutes post exhaustion (Biosen 5130, EKF-diagnostic, GmbH, Barleben, Germany).

### Statistical analysis and multivariate modeling

Distribution of data at initial testing were analyzed with Shapiro-Wilk W Goodness-of-Fit test and found, with a few exceptions due to single outliers (eosinophils, EVF, MCHC, bilirubin and transferrin among men), to be normal (p>0.05). For analysis of performance and hematological variables, repeated measures multivariate analysis of variance (MANOVA) was applied. The sphericity Chi-square test checked within-subject effects. If significant, the F-test was adjusted (denoted as ε) for degrees-of-freedom according to Greenhouse and Geisser [48], and one-way ANOVA used to compared groups at specific time points. Mean and standard deviation (SD) of data is presented unless specifically noted.

Numerous statistical strategies have been proposed to investigate hematological data in response to autologous blood transfusion [38, 41, 49–51], EPO injections [47, 52] and altered erythropoiesis [53]. Published approaches have also been questioned [36]. As a novel approach, we have applied an established multivariate regression method, with pattern recognition using Principal Component Analysis (PCA), Orthogonal Projections of Latent Structures (OPLS) [54], and its discriminant analysis OPLS-DA [55]. These methods are common in bioinformatics, and have previously been used by our group to discriminate clean human skeletal muscle samples from users of anabolic steroids [56], as well as establishing physical requirements for Swedish firefighters [57]. The strategy efficiently reveals possibly clusters and distinctions between the Control and Transfused subjects. All data was mean centered and scaled to unit variance (UV) prior to analysis. An unsupervised PCA was performed (if R^2^ = 1, the model explains 100% of the variation in the data). OPLS and OPLS-DA models were used to separate Control from Transfused (R^2^) and for predictive power (also called cross-validation) of the model (Q^2^). R^2^ and a Q^2^ > 0.60 were deemed valid. In OPLS, X represents the regressor variables (hematological variables) and Y represents the response variables (Control and Transfusion). VIP (Variable Important for Projection) summarizes the importance of the variables both to explain X and to correlate to Y. VIP is normalized, and the average squared VIP value is 1, thus VIP > 1 indicates that the variable is important for the projection, and values lower than 0.5 indicates that the variable is unimportant for the projection. Significance is set by Rules 1, 2 and 3; Q^2^ > Limit (indicated as R1, R2 and R3 in Results, where the Limit depends on number of components for PCA and Y-variables for OPLS. For further reading on the statistical methods we refer to [54, 58, 59] and www.umetrics.com. Data were analyzed using JMP 11.2.0 (SAS Institute Inc., USA) and SIMCA 14.0 (MKS AB, Sweden).

## Results

**Fig 2:**
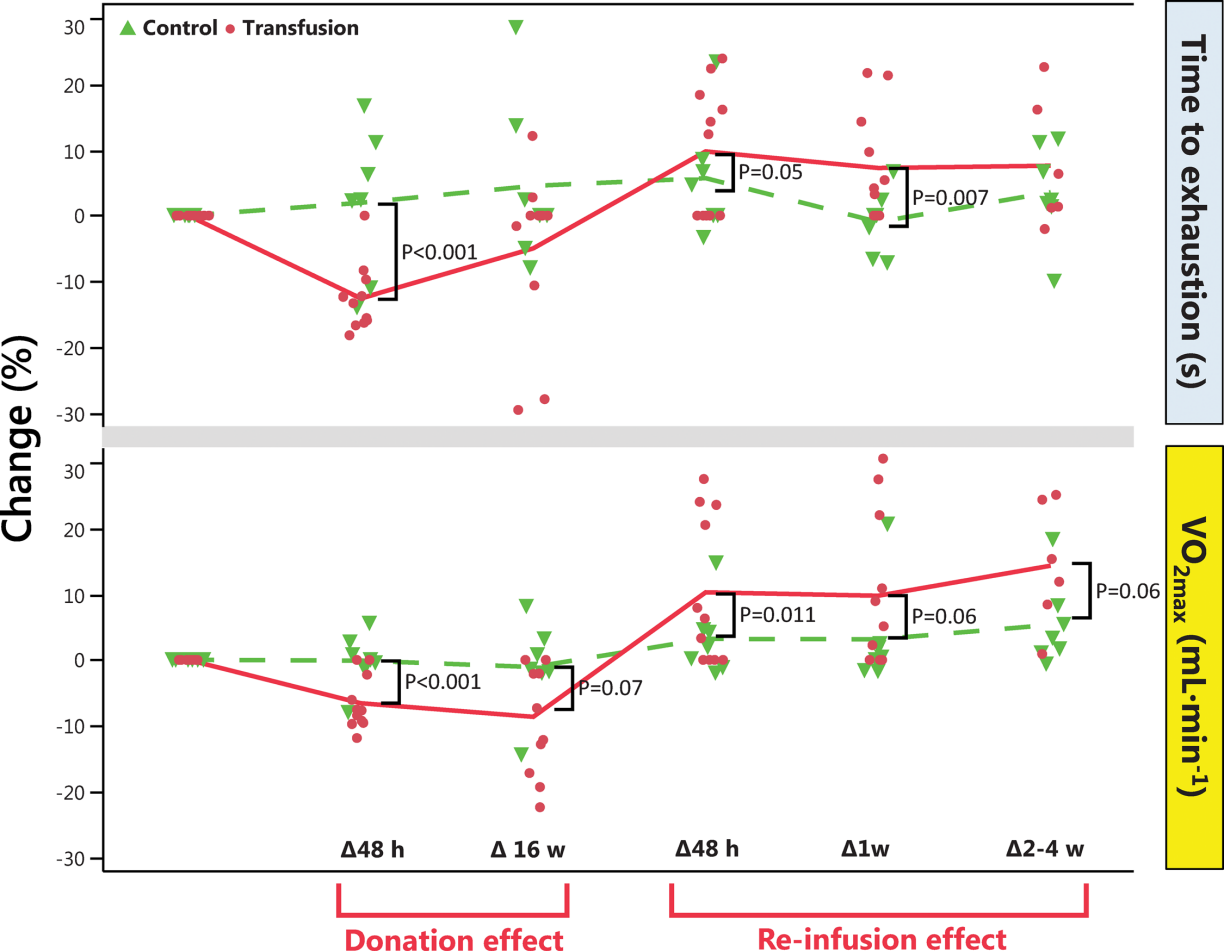
Change in performance and VO_2max_ (mL·min^-1^) with blood donation and RBC re-infusion in Part A of the Study. Data is presented as percent change from baseline, with a line connecting means. The graph shows the effects of blood donation and RBC re-infusion on repeated testing for time to exhaustion and VO_2max_ in the Transfusion group, and changes over time with repeated testing only in the Control group. For effects of blood donation baseline was set to Week 0, and changes in data on VO_2max_ and time to exhaustion calculated to 48 h and 16 weeks after donation. For effects of RBC re-infusion baseline was set to Week 16. MANOVA demonstrated a difference over time between groups, and differences between groups at each time point (oneway ANOVA, p<0.1) are indicated by brackets. Changes at week seven is not shown, and results from tests weeks 19–21 collated. Control as dashed line (-—-), Transfusion as solid line (—). Difference between groups over time investigated by repeated measures MANOVA was significant (F = 12, p-value by Greenhouse-Geisser ε = 0.03), and one-way ANOVA at each time-point subsequently calculated.

**Fig 3:**
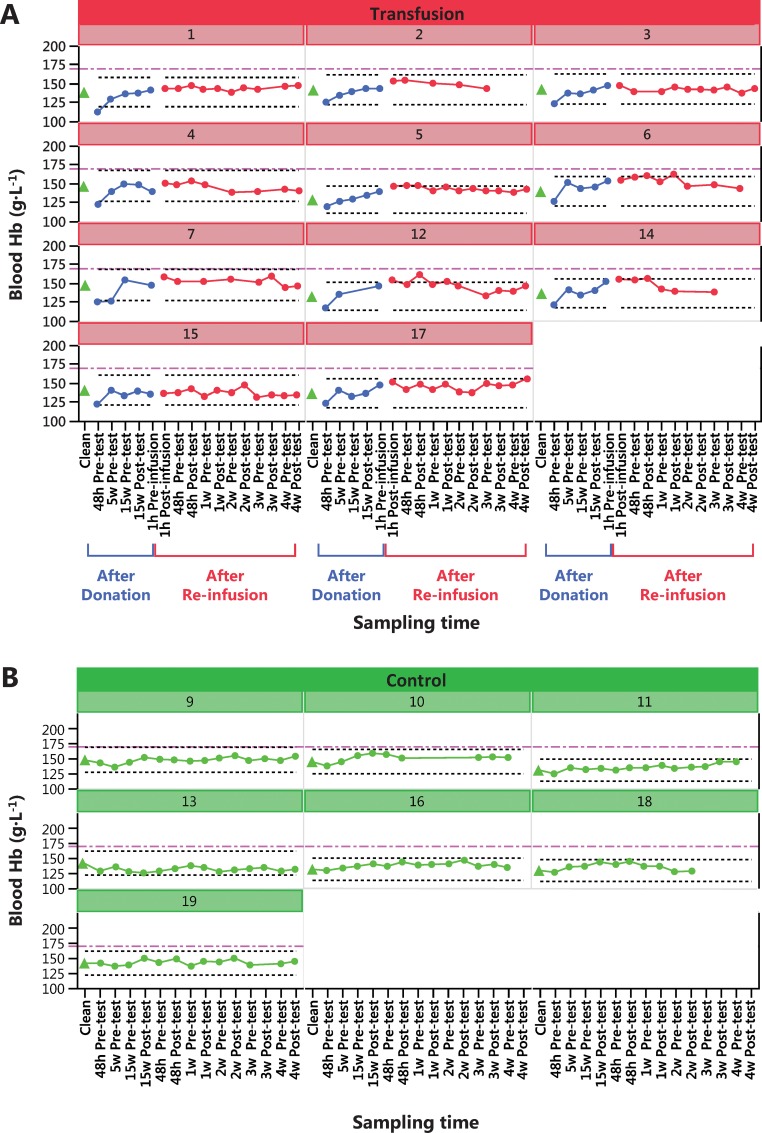
Part A of the Study. **Individual blood [Hb] (g·L**^**-1**^**).** Results from analysis of 161 blood samples from transfused men (N = 11, Nr 1–17) in Panel A, and 93 samples from control men in Panel B (N = 7, Nr 9–19). All samples were taken in parallel during 22 weeks (Fig 1). Transfused subjects donated two units (900 mL) blood and were re-infused with the washed, cryopreserved RBC fraction at week 17 (Fig 1). Control subjects were sampled and tested at the same time intervals, except no sample was taken at one hour before and after the RBC reinfusion. For reference, a dashed black line at [Hb] ± 15% from the first sample (noted as Clean in both groups) is suggested to be the individual upper and lower limits for indication of autologous blood doping [41]. Upper limit for [Hb] set at 170 g·L^-1^ for men by some sport federations such as FIS, is indicated by dash-dot purple lines.

**Fig 4:**
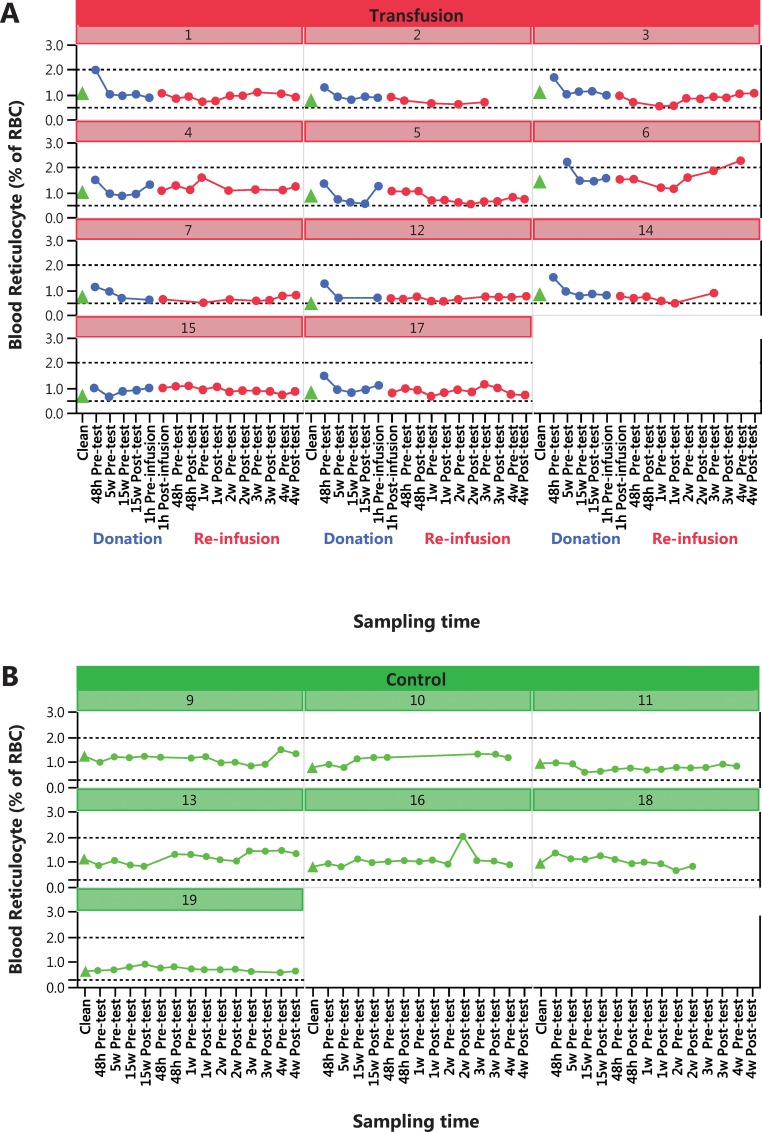
Part A of the Study. **Individual blood reticulocyte count per RBC (%).** Results from analysis of 161 blood samples from transfused men (N = 11, Nr 1–17) in Panel A, and 93 samples from control men in Panel B (N = 7, Nr 9–19). All samples were taken in parallel during 22 weeks (Fig 1). Transfused subjects donated two units (900 mL) blood and were re-infused with the washed, cryopreserved RBC fraction at week 17 (Fig 1). Control subjects were sampled and tested at the same time intervals, except one hour before and after the RBC reinfusion. For reference, a dashed black line at 0.5% and 2% is suggested to be the individual upper and lower limits for indication of autologous blood doping [60].

**Fig 5:**
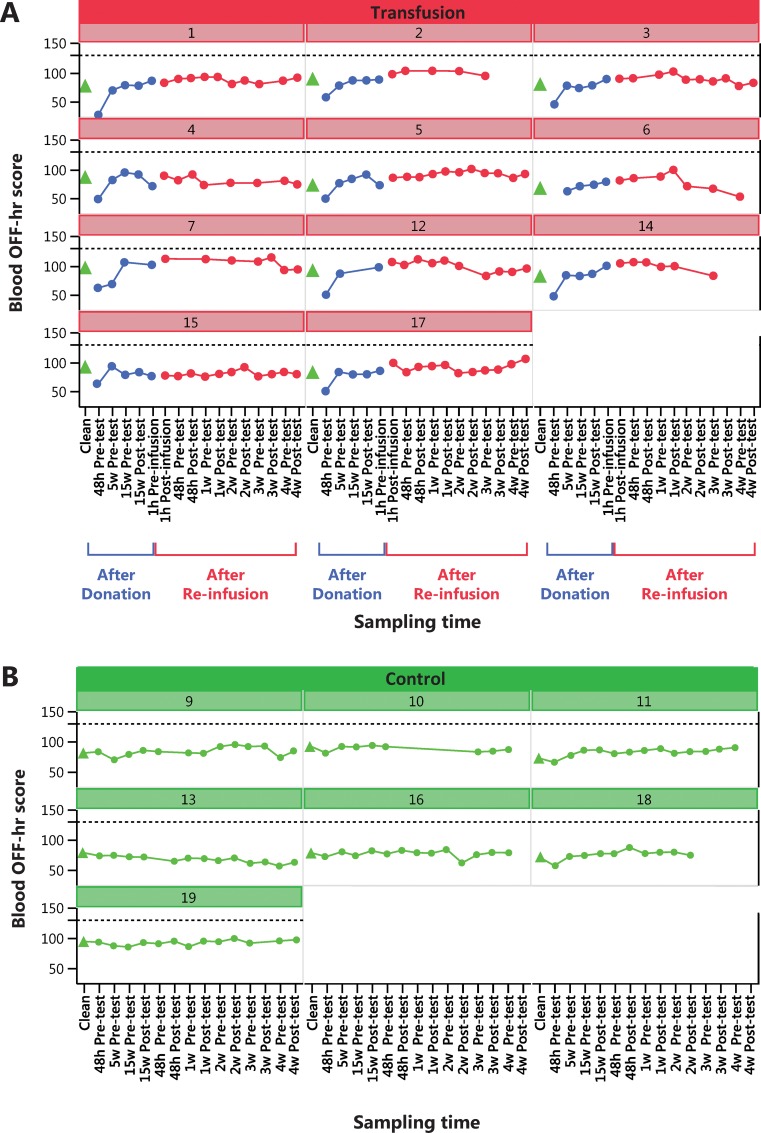
Part A of the Study. **Individual blood OFF-hr score.** Results from analysis of 161 blood samples from transfused men (N = 11, Nr 1–17) in Panel A, and in Panel B 93 samples from control men (N = 7, Nr 9–19). All samples were taken in parallel during 22 weeks (Fig 1). Transfused subjects donated two units (900 mL) blood and were re-infused with the washed, cryo-preserved RBC fraction at week 17 (Fig 1). Control subjects were sampled and tested at the same time intervals, except one hour before and after the RBC reinfusion. For reference, a dashed black line at OFF-hr = 129 (OFF-hr = $\text{Hb}-60\sqrt{Ret\left(\%\right)}$) is one suggested limits for indication of blood doping with EPO [47] but used by others for investigation of blood manipulation in general.

**Fig 6:**
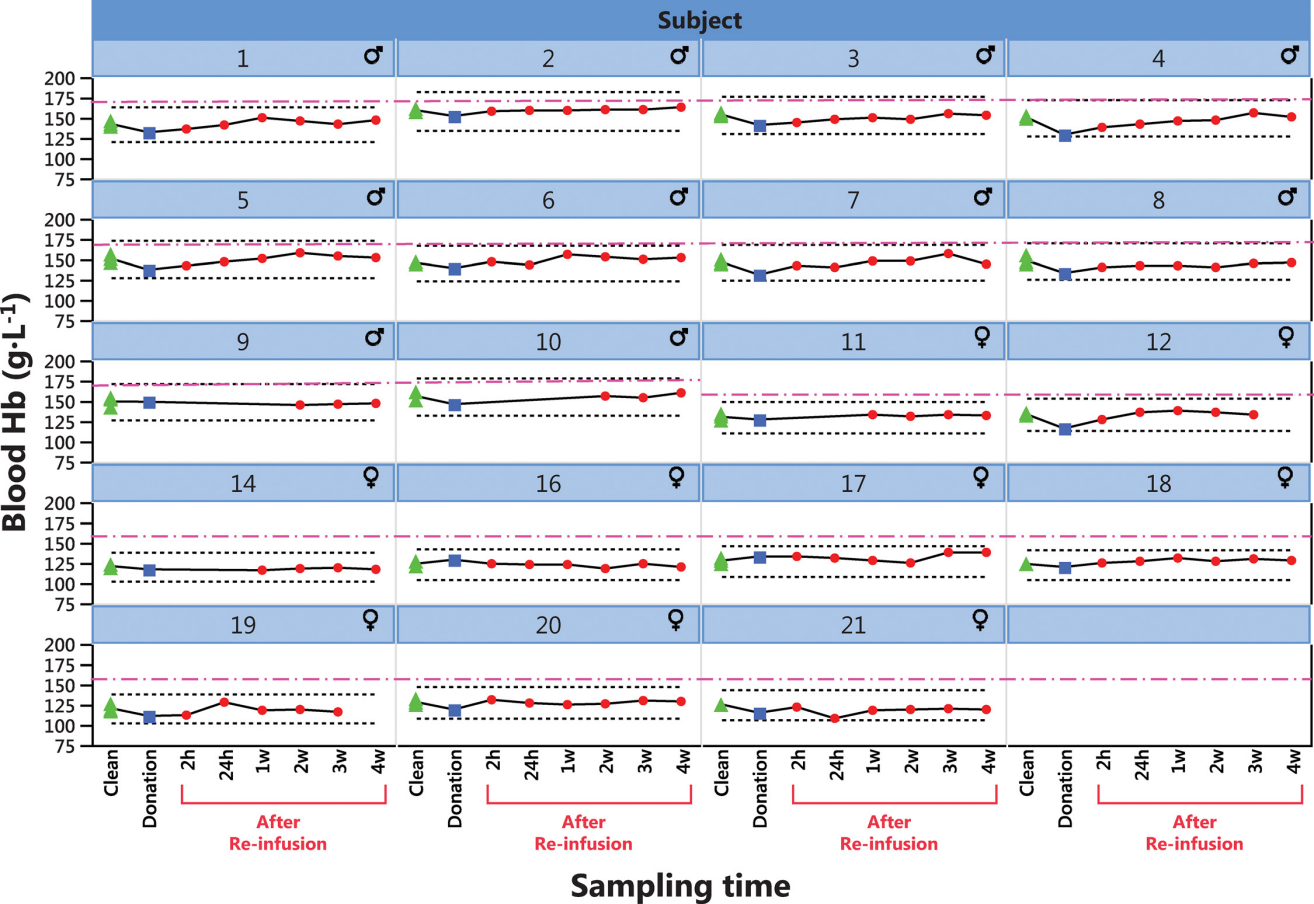
Part B of the Study. **Individual blood [Hb] (g·L**^**-1**^**) from ten men and nine women during a ten week period before and after autologous blood transfusion.** One (women) and two (men) units (450 mL each) whole blood was withdrawn, with re-infusion of the washed, cryo-preserved RBC fraction five weeks later (Fig 1). A total of 190 samples are included from women (N = 80) and Men (N = 110). Over time, samples are group into Clean (N = 57), Donation (N = 19) and Doped (N = 114. Upper limit for [Hb] set by some some sport federations, such as FIS, for women at 160 g·L^-1^ and for men a 170 g·L^-1^ is indicated by dash-dot purple lines. ▲(green) Clean (triplicate samples), ■ (blue) After blood donation, ● (red) After RBC re-infusion.

**Fig 7:**
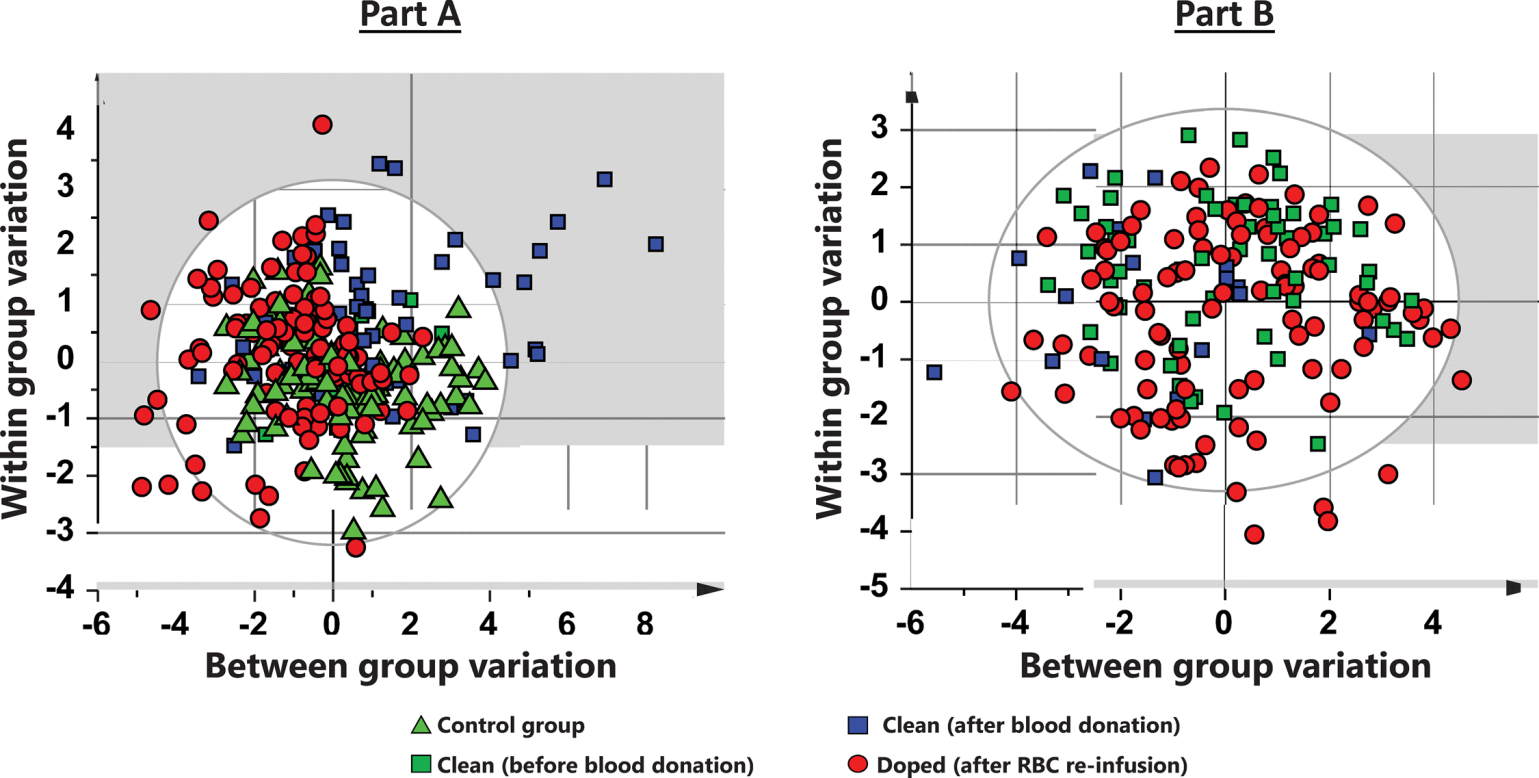
**Part A of the Study and Part B of the Study. PCA overview comparing scores scatter plot of Control, Clean, Donated and Transfused and subjects.** Observations close to each other are more similar than observations distant from each other. Each marker represents the sum of all hematological variables. **Panel A** is data from 323 blood samples taken from the Control and Transfusion groups, over a period of 21 week (Fig 1). A PCA analysis including all data from nine hematological variables (Table 5) with N = 323 blood samples and X = 2272 data points (individual hematological data) indicated a separation between Control and Transfusion groups (R^2^X = 0.56; Q^2^ = 0.38; significant by R1). **Panel B** is data from 210 blood samples taken over 10 weeks (Fig 1) where Clean samples are taken before blood donation (green squares), Post donation samples are indicated by blue squares and Doped samples by red circles. R ^**2**^ = 0.60, and Q ^**2**^ = 0.31. Clean samples from the Control group (▲); Transfusion group before donation (■), Transfusion group after donation (■), Transfusion group after RBC re-infusion (●). Ellips indicates 95% CI.

**Fig 8:**
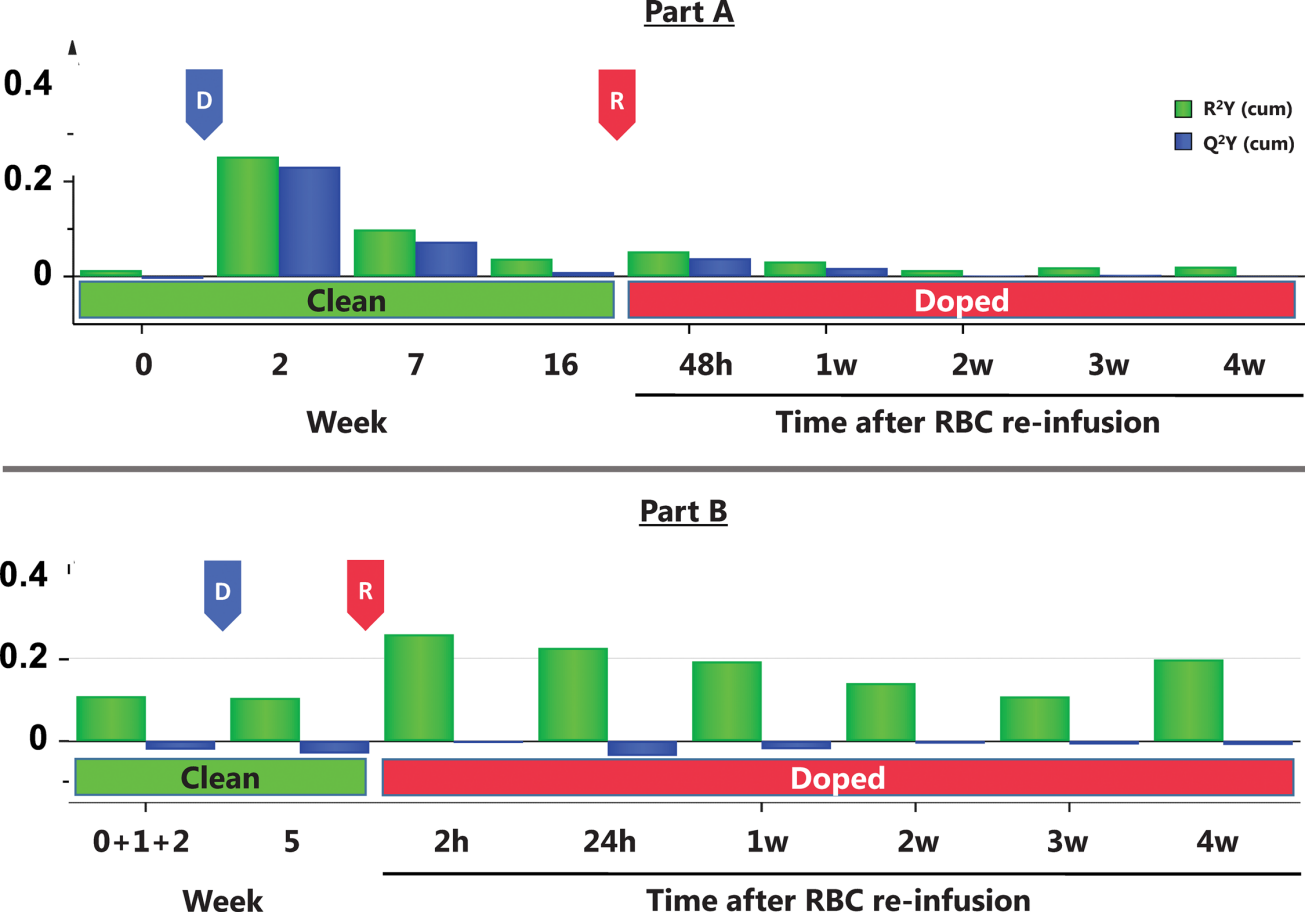
**Part A of the Study and B. OPLS X/Y overview plot of the effect of blood donation and RBC re-infusion over time.** The OPLS X/Y overview plot displays the individual cumulative Regression (R^2^) and Prediction (Q^2^) for every time point of blood sampling in Part A of the Study and B. **Part A of the Study:** R^2^X = 0.60; Q^2^ = 0.04. **Part B of the Study:** R^2^X = 0.49; Q^2^ = -0.01. Neither part (A and B) of the study reached a significant separation of Clean and Doped subjects based on hematological variables. D; Blood donation, R; RBC re-infusion.

**Fig 9:**
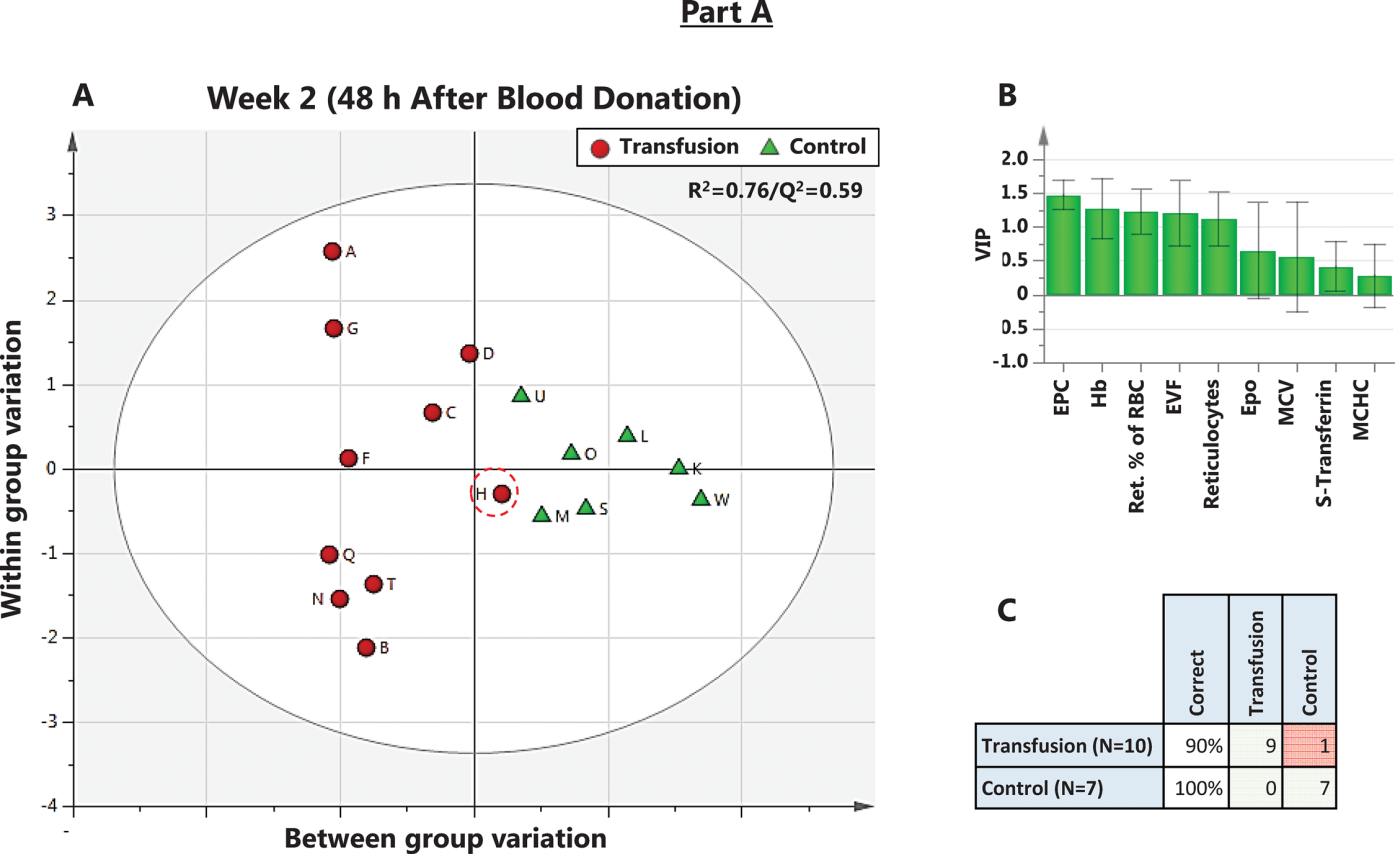
Part A of the Study. **An OPLS-DA analysis at 48 h after blood donation.** A) Scores Scatter plot showing separation of Control and Transfusion groups 48 h after blood donation. This plot shows how the modeled observations in X space are situated with respect to each other. Observations close to each other are more similar than observations distant from each other. Regression (R ^**2**^) = 0.76, and prediction by cross-validation (Q ^**2**^) = 0.59. B) VIP rank for included variables. C) Misclassification table showing one incorrectly predicted Transfused (Subject H, circled in red) to the Control group (p < 0.001, Fisher’s exact probability test).

**Fig 10:**
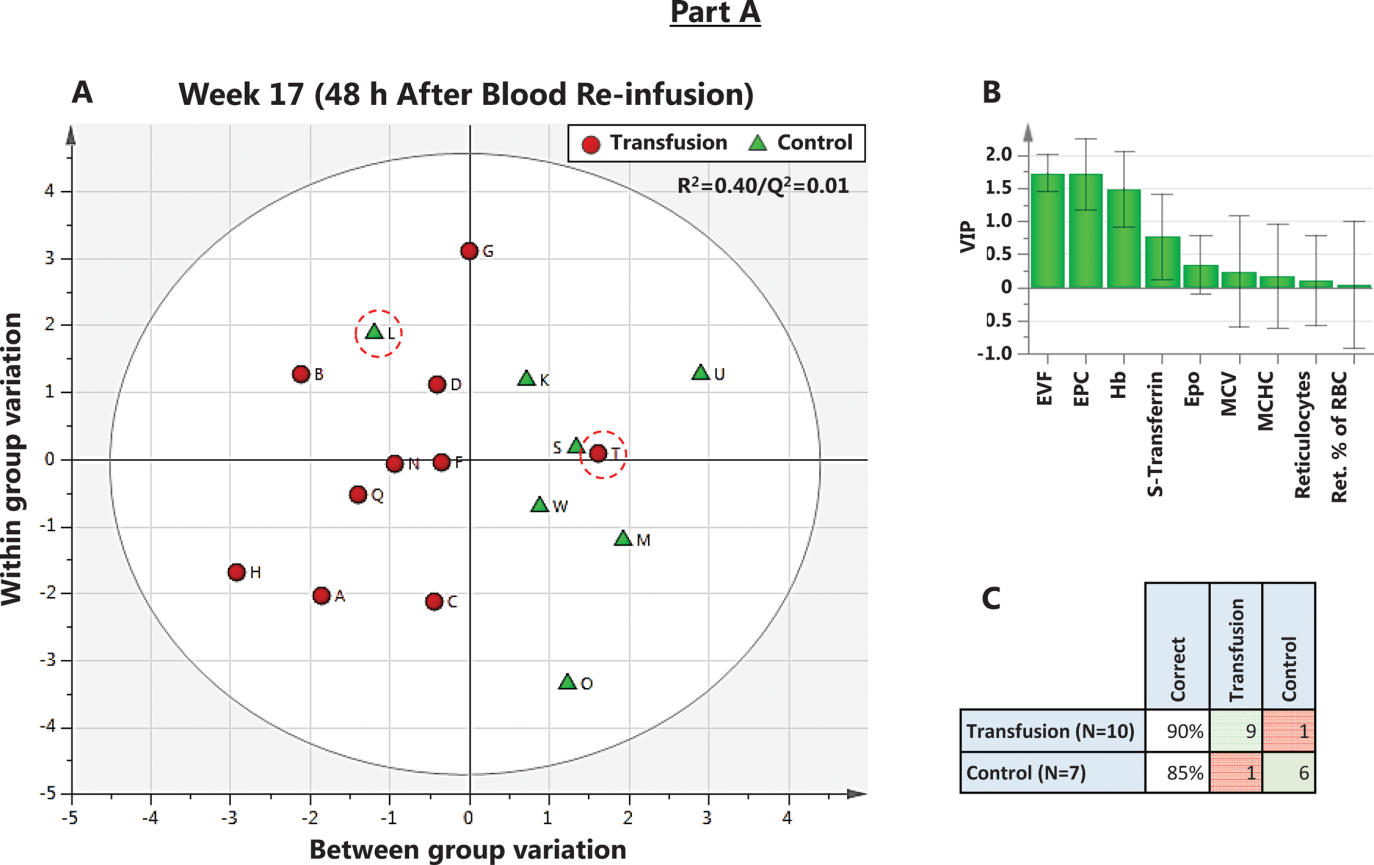
Part A of the Study. **An OPLS-DA analysis at 48 h after blood re-infusion.** A) Scores Scatter plot showing separation of Control and Transfusion groups 48 h after RBC re-infusion. Observations close to each other are more similar than observations distant from each other. Regression (R ^**2**^) = 0.40, and prediction by cross-validation (Q ^**2**^) = 0.01. B) VIP rank for included variables. C) Misclassification table showing one incorrectly predicted Transfused (Subject T) to the Control group, and one Control (Subjects L), predicted as Transfused (both circled in red; p = 0.004, Fisher’s exact probability test).

Change in physical performance in Part A of the Study (Table 4) was significantly different between the Transfusion and Control groups only when compared to individual baseline values (Fig 2). Group mean for hematological variable did in general not change in either part of the study (Part A of the study in Figs 3–5 and Table 5, Part B of the study in Fig 6 and Table 6). The first two principal components explain 68% and 68% of the total variance in hematological data for Part A of the Study and Part B of the Study, respectively. The score scatter plot of hematological data revealed no separation between the Clean and Transfused samples, neither in Part A of the Study nor in Part B of the Study (Fig 7). An OPLS and OPLS-DA analysis did not find significant models for separation of Control and post re-infusion samples any time point in either part of the study (Part A and Part B of the study in Fig 8 and Figs 9 and 10).

**Table 4:** Physical performance and maximal aerobic power as an effect of blood transfusion (Part A of the Study).

	Time to exhaustion (sec)		VO2max (ml·min-1)	
	Control	Transfusion	Control	Transfusion
**Baseline (N: C = 7/T = 9)**	382 ± 46	417 ± 59	4762 ± 535	4732 ± 499
**48h post donation (N: C = 7/T = 8)**	390 ± 68	342 ± 60	4761 ± 610	4315 ± 509
**5w post donation (N: C = 6/T = 9)**	378 ± 74	395 ± 55	4936 ± 549	4538 ± 501
**15w post donation (N: C = 6/T = 7)**	409 ± 92	389 ± 70	4914 ± 567	4159 ± 722
**48h post re-infusion (N: C = 7/T = 6)**	421 ± 76	454 ± 65	4859 ± 680	4660 ± 476
**1w Post re-infusion (N: C = 6/T = 6)**	412 ± 77	428 ± 74	4759 ± 595	4741 ± 377
**2w Post re-infusion (N: C = 6/T = 4)**	437 ± 94	405 ± 52	4910 ± 696	4660 ± 552
**3-4w Post re-infusion (N: C = 2/T = 5)**	-	384 ± 27	-	4615 ± 452

Mean ± SD. No significant difference from Control (p<0.05 by oneway ANOVA).—N = 2, no statistics possible.

**Table 5:** Part A of the Study. Hematological variables.

				Time								
				Clean	After donation			After RBC re-infusion				
Variable	MANOVA	Group	N	Week 0	Week 2	Week 7	Week 16	Week 17	Week 18	Week 19	Week 20	Week 21
**Transferrin (g·L**^**-1**^**)**	(Time*Group: F = 0.32, P = 0.24)	**Control**	7	2.3 ± 0.4	2.2 ± 0.3	2.3 ± 0.4	2.2 ± 0.3	2.2 ± 0.3	2.1 ± 0.2	2.1 ± 0.3	2.2 ± 0.3	2.4 ± 0.2
		**Transfusion**	10	2.3 ± 0.3	2.3 ± 0.2	2.6 ± 0.3	2.3 ± 0.2	2.4 ± 0.3	2.3 ± 0.3	2.3 ± 0.3	2.3 ± 0.2	2.3 ± 0.3
**Hemoglobin (g·L**^**-1**^**)**	(Time*Group: F = 21, P = 0.020)	**Control**	7	138+-7	132 ± 7	136 ± 4	138 ± 9	140 ± 10	138 ± 4	137 ± 9	140 ± 7	141 ± 8
		**Transfusion**	10	139 ± 6	121 ± 4*	136 ± 8	139 ± 8	148 ± 6	145 ± 5*	143 ± 6	142 ± 6	142 ± 4
**Erythrocytes (10**^**12**^**·L**^**-1**^**)**	(Time*Group: F = 22, P < 0.001)	**Control**	6	4.7 ± 0.2	4.7 ± 0.2	4.7 ± 0.2	4.8 ± 0.3	4.8 ± 0.3	4.7 ± 0.2	4.7 ± 0.4	4.8 ± 0.2	4.8 ± 0.3
		**Transfusion**	10	4.7 ± 0.3	4.2 ± 0.2*	4.6 ± 0.2	4.8 ± 0.4	5.1 ± 0.2*	4.9 ± 0.3	4.9 ± 0.3	4.8 ± 0.2	4.8 ± 0.2
**EVF (%)**	(Time*Group: F = 17, P = 0.020)	**Control**	6	41 ± 2	40 ± 2	41 ± 1	41 ± 2	41 ± 3	40 ± 1	40 ± 2	41 ± 1	41 ± 2
		**Transfusion**	10	41 ± 2	37 ± 1*	41 ± 2	42 ± -2	44 ± 2*	42 ± 1*	42 ± 1*	42 ± 1	41 ± 1
**MCV (fL)**	(Time*Group: F = 0.2, P = 0.01)	**Control**	7	86 ± 4	86 ± 4	87 ± 3	86 ± 3	86 ± 3	86 ± 3	86 ± 3	86 ± 3	86 ± 3
		**Transfusion**	10	88 ± 3	88 ± 4	90 ± 3	86 ± 3	86 ± 3	86 ± 2	86 ± 3	86 ± 3	86 ± 3
**MCHC (g·L**^**-1**^**)**	**(Time: F = 0.3, P < 0.001)**	**Control**	6	340 ± 6	332 ± 4	334 ± 2	335 ± 8	339±7	340 ± 4	340 ± 6	343 ± 8	345 ± 8
		**Transfusion**	9	338 ± 4	330 ± 7	329 ± 7	334 ± 8	338±7	343 ± 7	341 ± 7	343 ± 8	345 ± 7
**Reticulocytes (10**^**9**^**·L**^**-1**^**)**	**(Time: F = 7, P = 0.46)**	**Control**	6	43 ± 11	43 ± 9	43. ± 8	46 ± 12	48±12	45.5 ± 11	39 ± 8	47.3 ± 15	50 ± 16
		**Transfusion**	9	42 ± 12	64 ± 16	47 ± 22	43 ± 13	47±16	37 ± 19	42 ± 15	46 ± 18	50 ± 23
**Ret/Eryth (%)**	(Time*Group: F = 0.4, P = 0.03)	**Control**	6	0.9 ± 0.2	0.9 ± 0.2	0.9 ± 0.2	1.0 ± 0.2	1.0 ± 0.2	1.0 ± 0.2	0.8 ± 0.2	1.0 ± 0.3	1.1 ± 0.4
		**Transfusion**	10	0.9 ± 0.3	1.5 ± 0.4*	1.0 ± 0.4	0.9 ± 0.3	1.0 ± 0.3	0.8 ± 0.4	0.9 ± 0.3	1.0 ± 0.4	1.0 ± 0.5
**Erythropoietin (E·L**^**-1**^**)**	(Time*Group: F = 0.3, P = 0.005)	**Control**	6	11 ± 4	12 ± 4	10 ± 3	12 ± 5	12 ± 6	10 ± 4	9 ± 3	11 ± 5	13 ± 6
		**Transfusion**	8	13 ± 5	21 ± 8*	12 ± 3	13 ± 5	8 ± 4	10 ± 5	11 ± 5	12 ± 6	1 ± 6

Mean ± SD. Repeated measure MANOVA (with Greenhouse-Geisser ε adjusted p-value if test for sphericity is significant) given for each variable.

*Significant difference from Control (p<0.05 by oneway ANOVA).

F-value is given for effect of Time*Group unless. EVF; Erythrocyte Volume Fraction, MCV; Mean Corpuscular Volume (of erythrocytes), MCHC; Mean Corpuscular Hemoglobin Concentration (of erythrocytes).

**Table 6:** Part B of the Study. Hematological variables.

			Time							
			Clean	After donation	After RBC re-infusion					
			Weeks 0, 1, 2	Week 5	Week 5 +2h	Week 5 +24h	Week 6	Week 7	Week 8	Week 9
Variable	MANOVA	Sex	N = 52	N = 19	N = 15	N = 15	N = 17	N = 19	N = 19	N = 17
**Transferrin (g·L**^**-1**^**)**	**Time: F = 8.9, P = 0.0.03**	**M+W**	2.4 ± 0.3	2.6 ± 0.3	2.5 ± 0.3	2.6 ± 0.4	2.7 ± 0.3	2.7 ± 0.3	2.7 ± 0.3	2.6 ± 0.3
**Iron (μmol·L**^**-1**^**)**	**Time: F = 0.004, P = 0.88**	**M+W**	19 ± 7	15 ± 6	18 ± 6	16 ± 9	19 ± 9	18 ± 11	17 ± 6	20 ± 6
**Bilirubin (μmol·L**^**-1**^**)**	**Time: F = 0.04, P = 0.58**	**M+W**	14 ± 5	13 ± 4	17 ± 5	16 ± 7	15 ± 7	14 ± 6	15 ± 7	15 ± 6
**Hemoglobin (g·L**^**-1**^**)**	Sex*: F = 4.9, P<0.0001	**M**	148 ± 8†	137 ± 8†	143 ± 7†	145 ± 6†	148 ± 8†	148 ± 8†	150 ± 8†	150 ± 8†
		**W**	126 ± 5	120 ± 8	125 ± 7	126 ± 9	124 ± 7	123 ± 6	126 ± 8	125 ± 8
**Leukocytes (10**^**9**^**·L**^**-1**^**)**	**Time: F = 1.7, P = 0.30**	**M+W**	6 ± 1	5 ± 1	5 ± 1	6 ± 1	6 ± 1	6 ± 1	6 ± 1	6 ± 1
**Neutrophils (10**^**9**^**·L**^**-1**^**)**	**Time: F = 1.6, P = 0.70**	**M+W**	3 ± 1	3 ± 1	3 ± 1	3 ± 1	3 ± 1	3 ± 1	3 ± 1	3 ± 1
**Lymphocytes (10**^**9**^**·L**^**-1**^**)**	**Time: F = 0.8, P = 0.89**	**M+W**	2 ± 1	2 ± 1	2 ± 0	2 ± 1	2 ± 1	2 ± 0	2 ± 1	2 ± 1
**Monocytes (10**^**9**^**·L**^**-1**^**)**	**Time: F = 5.9, P = 0.24**	**M+W**	0.5 ± 0.2	0.5 ± 0.2	0.5 ± 0.2	0.5 ± 0.2	0.5 ± 0.2	0.5 ± 0.2	0.5 ± 0.2	0.5 ± 0.1
**Eosinophils (10**^**9**^**·L**^**-1**^)*	** Time: F = 3.8, P = 0.23**	**M+W**	0.2 ± 0.1	0.2 ± 0.1	0.1 ± 0.1	0.2 ± 0.1	0.2 ± 0.1	0.2 ± 0.1	0.2 ± 0.1	0.2 ± 0.1
**Basophils 10**^**9**^**·L**^**-1**^)*	** No statistics possible**	**M+W**	0.1 ± 0	0.1 ± 0	0.1 ± 0	0.1 ± 0	0.1 ± 0	0.1 ± 0	0.1 ± 0	0.1 ± 0
**Erythrocytes (10**^**12**^**·L**^**-1**^**)**	Sex*: F = 2.4, P = 0.0004	** M**	5.0 ± 0.3†	4.7 ± 0.3†	4.8 ± 0.2†	4.9 ± 0.2†	5.0 ± 0.2†	5.0 ± 0.3†	5.1 ± 0.3†	5.1 ± 0.3†
		**W**	4.3 ± 0.2	4.1 ± 0.4	4.2 ± 0.3	4.3 ± 0.4	4.2 ± 0.3	4.2 ± 0.3	4.3 ± 0.3	4.3 ± 0.4
**Thrombocytes (10**^**9**^**·L**^**-1**^**)**	**Time: F = 9.9, P = 0.023**	**M+W**	220 ± 35	210 ± 32	208 ± 37	221 ± 38	231 ± 44	233 ± 43^#^	230 ± 41”	224 ± 41
**EVF (%)**	Sex*: F = 3.3, P<0.0001	**M**	43 ± 2†	40 ± 2†	42 ± 2†	43 ± 2†	44 ± 3†	44 ± 3†	44 ± 3†	44 ± 2†
		**W**	38 ± 1	37 ± 3	38 ± 2	38 ± 3	38 ± 2	38 ± 2	38 ± 2	38 ± 3
**MCV (fL)**	**Time: F = 22, P<0.05**	** **	88 ± 5	88 ± 5	88 ± 5	89 ± 5	89 ± 5	88 ± 4	88 ± 5	87 ± 3
**MCH (pg)**	**Time: F = 0.42, P = 0.004**	** **	30 ± 2	29 ± 2	30 ± 2	30 ± 2	30 ± 1	29 ± 2	30 ± 2	29 ± 1
**MCHC (g·L**^**-1**^**)**	**Time: F = 39, P = 0.02**	**M**	342 ± 8†	342 ± 9†	345 ± 9†	342 ± 8†	341 ± 11†	340 ± 9†	344 ± 7†	342 ± 7†
		**W**	328 ± 6	323 ± 9	330 ± 7	325 ± 8	327 ± 6	324 ± 11	330 ± 6	325 ± 10

Mean ± SD. Repeated measure MANOVA (with Greenhouse-Geisser ε adjusted p-value if test for sphericity is significant) given for each variable. Time indicate significant change over time, men and women combined.

Time*Group indicate a difference in response between men and women. Sex indicate a difference between men and women, independent of Time.

^#^Significant difference from Clean at this time point (p<0.05 by oneway ANOVA).

†Significant difference between Men and Women (p<0.05 by oneway ANOVA).

EVF; Erythrocyte Volume Fraction, MCV; Mean Corpuscular Volume (of erythrocytes), MCH; Mean Corpuscular Hemoglobin Mass (of erythrocytes), MCHC; Mean Corpuscular Hemoglobin Concentration (of erythrocytes). M; Men, W; Women. If a significant difference between men and women was observed, data was split for effect over Time*Group. Statistics not possible due to repeated 0 value for basophils.

### Aerobic capacity and physical performance

In Part A of the Study, the aerobic work capacity and physical performance were tested nine times, the result shown in Table 4. Due to illness, injuries and other factors, some subjects failed to perform all tests. Thus, performance data is collated for week 2–4 in Fig 2, including only the best performance for subjects completing more than one test. A significant Time*Group effect was observed for both running time to exhaustion (F = 4.9, p < 0.001) and VO_2max_ (F = 4.7, p < 0.001).

Compared to Control, running time to exhaustion was decreased after blood donation, and increased 48 h after RBC re-infusion (Fig 2). Blood donation decreased VO_2max_ for 16 week, and RBC re-infusion increased VO_2max_ for 2–4 weeks, as compared to Control. Compared to initial values, mean value for groups did not changed significantly, neither for running time to exhaustion, nor for VO_2max_ (Table 4). Individual variations in the response to both donation and re-infusion is seen as outliers in Fig 2. Blood pressure, lactate and heart rate did not change significantly between groups over time, and only initial resting values presented (Tables 2 and 3).

### Hematological data

Hematological data was collected at 17 occasions (Fig 1) for the Transfusion group and at 15 occasions for the Control group, both before and after exercise testing. Only samples taken at rest are included in this study, giving nine samples from each individual (Tables 5 and 6). The acute (within 2 h) hemodynamic effects of exercise and RBC re-infusion are thus not discussed.

Over time (analyzed by repeated measures MANOVA), there was a significant difference between Control and Transfused groups for Hb, EPC, EVF, MCV, Ret. % of RBC (Fig 6) and Erythropoietin in Part A of the Study (Table 5). In Part B of the Study, the response of transfusion differed between men and women in EPC, [Hb] and EVF, while changes over time only was observed for MVC, MCH, MCHC and transferrin (Table 6).

In Part A of the Study, and In conjunction with the repeated testing procedures of athletes dictated by WADA, individual changes over time is displayed for blood [Hb] (Fig 3), blood reticulocyte count per RBC (%) (Fig 4) and calculated OFF-hr score (Fig 5). Data from Part B of the Study is plotted by individual in Fig 6, where a blood [Hb] baseline was established by three samples taken with one week intervals, displayed collectively as Clean. Boundaries for abnormal variations according to published studies are inserted. Only a few samples are ever outside these limits; [Hb] for subjects 6, 12 and 14, subject 6 for reticulocytes and no sample for the OFF-hr score.

### Multivariate models for separation of transfused form control samples

#### Part A of the Study

A PCA analysis (Fig 7) including all data from nine hematological variables (Tables 5 and 6) with 323 observations (blood samples; N) and 2272 data points (individual hematological data; X) indicated a separation between Control and Transfusion groups (R^2^X = 0.56; Q^2^ = 0.38; significant by R1). Using the same data, and setting each of nine time point as Y, yielded no significant OPLS model (R^2^X = 0.60; Q^2^ (cum) = 0.04) at any time (Fig 8). These analyses indicate a lack of correlation between hematological variables and categorized group. In the over-all OPLS-DA model, setting Control and Transfusion as Y, a regression of R^2^X (cum) = 0.48 and a predictive power of Q^2^ (cum) = 0.06 was reached. This model predicts 70% correctly classified Transfusion samples and 30% false positive (41 of 136 Control samples predicted to the Transfusion group). The over-all correct classification was 51%. Removing variables with low scores in the model did not improve classification.

#### Part B of the Study

A PCA analysis (Fig 7) including data from all 16 hematological variables in Table 6 (N = 210, X = 2881) indicated a separation between Control and Transfusion groups (R^2^X = 0.60; Q^2^ = 0.31; significant by R1). Using the same data, and setting each time point as Y (Fig 8), yielded no significant OPLS model (R^2^X = 0.49; Q^2^ (cum) = -0.01). These analyses indicate a lack of correlation between hematological variables and categorized state of the subjects. In the over-all OPLS-DA model, setting Y as each of three subjects states (Clean, Transfused and Doped), a regression of R^2^X (cum) = 0.39 and a predictive power of Q^2^ (cum) = 0.01 was reached. This model predicts 78% correctly classified Transfusion samples and 86% false positive (49 of 63 Clean samples set to the Transfusion group). The over-all correct classification was 52%. Removing variables with low scores in the model did not improve classification. Separation of Men and Women based on hematological variables was significant (R^2^X = 0.40; Q^2^ (cum) = 0.70, R1), but no significant models for each sex separately could be created (R^2^X = 0.49/0.48; Q^2^ (cum) = -0.01/0.02) for Men/Women respectively.

In Part A of the Study, the best model for separation of Clean and Transfused subjects based on hematological variables was reached at week 2, 48 h post donation (R^2^ = 0.76; Q^2^ = 0.59, R1, Fig 9), with low-scoring variables (VIP < 1) omitted (Fig 9B). Prediction by cross-validation gave one false positive, circled in red in Fig 9C (Fishers’ exact test p<0.0001). When the same procedure was repeated for samples collected 48 h after RBC re-infusion (Fig 10), the model (R^2^ = 0.40/Q^2^ = 0.10; R1) predicted one false positive and one false negative (Fig 10C). In Table 7, a misclassification matrix from OPLS-DA models at each time-point indicates 14 of 56 Control samples as Transfused, giving 25% false positives. Of 77 Doped samples, 17 (22%) was misclassified as Clean or No Class, leaving 82% of all Doped samples correctly classified as Doped. Similar results were achieved in Part B of the Study (Table 8).

**Table 7:** Part A of the Study. Misclassification table.

Week	Time point	R^2^/Q^2^	Group	Correct (%)	Transfusion	Control	No class	P
**2**	48 h Post Donation	0.76/0.59	**Transfusion**	90	9	**1**	0	<0.01
			**Kontroll**	100	0	7	0	
**7**	5 w Post Donation	0.56/0.21	**Transfusion**	80	8	**2**	0	0.05
			**Kontroll**	71	**2**	5	0	
**16**	15 w Post Donation	0.60/-0.46	**Transfusion**	70	7	**3**	0	0.48
			**Kontroll**	43	**4**	3	0	
**17**	48 h Post Re-infusion	0.55/0.10	**Transfusion**	90	9	**1**	0	<0.01
			**Kontroll**	86	**1**	6	0	
**18**	1 w Post Re-infusion	0.56/0.20	**Transfusion**	80	8	**2**	0	-
			**Kontroll**	86	0	6	**1**	
**19**	2 w Post Re-infusion	0.55/-0.08	**Transfusion**	80	8	**1**	**1**	-
			**Kontroll**	43	3	3	**1**	
**20**	3 w Post Re-infusion	0.45/-1.31	**Transfusion**	70	7	3	0	-
			**Kontroll**	57	**2**	4	**1**	
**21**	4 w Post Re-infusion	0.41/-0.88	**Transfusion**	70	7	**1**	2	-
			**Kontroll**	57	**2**	4	**1**	

P; Fisher’s probability.

- Not calculated with 3 groups (Transfusion, Control and No class).

N = 136. False positive ratio = 14/56 (25%), False negative ration 14/80 (18%). **Bold** number indicate wrong class.

**Table 8:** Part B of the Study. Misclassification table.

	N	Correct	Clean	Donation	Doped	No class
**Clean**	63	14%	9	0	**49**	**5**
**Donation**	21	0%	2	0	**18**	**1**
**Doped**	126	79%	**5**	0	99	**22**
**Total**	210	51%	16	0	166	28

N = 210. False positive ratio = 49/63 (86%), False negative ratio 5/129 (11%). **Bold** number indicate wrong class.

## Discussion

The main purpose of this study was to investigate if hematological variables can be used in multivariat statistical models to detect autologous blood transfusion.

### Physical performance

Aerobic work capacity (VO_2max_) and running performance decreased significantly compared to individual baseline after donation of 2x450 mL whole blood, and was for most subjects nor recovered after 15 weeks. After RBC re-infusion at this time, performance increased by ~20% (Fig 2), in some individuals increased for 3–4 weeks. Changes in VO_2max_ and performance was not significantly correlated (R^2^ < 0.5). Large individual variations in response to blood transfusion partially explaining the lack of correlation. In Fig 2 these variations are apparent, with a few subjects decreasing with up to 30% in performance 16 weeks after blood donation, testifying the hardship with training after donation of two units of blood. Comparing changes in in the Control and Transfusion groups (Fig 2) demonstrates an inter-individual difference in response to both exercise and blood donation/re-infusion. Not all individuals increase in performance after RBC re-infusion, even if VO_2max_ is increased. The effect of repeated testing is apparent in the Control group, with subjects increasing up to 30% in performance on the forth test.

Acute and chronic decrease in physical performance capacity with anemia is well documented by Ekblom [4, 23] and Celsing [61–63], and can be explained by lower blood volume [64] and decreased Hb_mass_ [6, 64], but not blood [Hb] [64]. Present data supports these findings, because blood Hb concentration is normalized 5 weeks after blood donation, while VO_2max_ is not recovered until 48 h after RBC re-infusion. Also, there is a lack of correlation between changes in blood [Hb] and VO_2max_ to performance in the Transfusion group (R^2^ < 0.1; p > 0.1). The prolonged decrease in VO_2max_ compared to individual baselines (Fig 2) can therefore not be explained by altered [Hb]. It is suggested that an acute decrease in VO_2max,_ as well as performance after blood donation results in a lower training capacity during weeks to follow, further decreasing performance.

Earlier studies [5, 23, 30, 31, 65] investigating the performance after blood re-infusion, also shows an increase in both VO_2max_ and performance directly after re-infusion [28]. In the study by Buick [5], VO_2max_ was elevated for at least 17 weeks post re-infusion, while running time to exhaustion increased for up to 7 days only, mimicking the present results (Fig 4). Increased VO_2max_ following re-infusion of RBC has been explained by the increased oxygen carrying capacity of the blood, due to an increased number of RBC, [Hb], blood volume, total Hb_mass_ [23] and Q [22]. The concentration of RBC (EPC in Table 1), [Hb] and hematocrit (EVF in Table 5) did not change in parity with VO_2max_ and performance, and were at no time correlated. Thus, blood volume, O_2_ muscle extraction and Q_max_ are remaining factors explaining the substantial (15–25%) increase in VO_2max_. Physical performance may also be influenced by several other factors, including but not limited to; motivation, local and systemic anaerobic capacity and effect, especially among non-elite subjects as in the present study. Thus, the lack of maintained enhanced performance may have a multitude of explanations, while the physiological effects of blood re-infusion cannot be masked.

### Hematology

To address the issue of discriminating clean athletes from athletes doped by autologous blood transfusion of cry-preserved RBCs, two separate study designed were completed (Part A of the Study and Part B of the Study in Fig 1). The result of the hematological measurements showed significant differences between Control and Transfusion groups for mean values of [Hb], EPC, EVF, reticulocytes as % of RBC and EPO (Table 5), and also with blood transfusion over time in Part B of the Study, where erythrocytes, thrombocytes, MCV, MCH and MCHC changed significantly (Tables 5 and 6). Data from both Part A of the Study and B demonstrate a rather rapid hemodynamics of healthy males and females, where plasma expansion and RBC filtration in the spleen [66] will, at least under present conditions, return hematological variables to normal more rapid than previously reported [41, 50, 67]. The use of hematological variables to detect autologous blood doping has been applied for almost 30 years [39], and various algorithms developed [47, 49–51, 60, 68]. The presently observed short-lived alterations in all hematological variables, including EPO, suggest that any indicator of blood transfusion requires blood sampling close in time to either blood donation or RBC re-infusion. This holds true also for other suggested markers of autologous blood transfusion [69, 70]. With the known confounding factors [40] concerns regarding the use of these variables for doping detection has already been raised [36].

Viewing individual changes over time (Hb, reticulocytes and OFF-hr) in response to blood donation and RBC re-infusion (Figs 3–6), it is apparent that few data points ever deviate from normal variation, and also that observed effects over time are individually different. Individual limits have been suggested [39], such as ±15% in [Hb] [41], 0.5% and 2% for percent reticulocyte [60], and an OFF-hr above 129 [47], but few samples in the present study are ever outside these limits, despite re-infusion of up to 400 mL of RBCs. Also, the 170 g·L^-1^upper limit for [Hb] in men, and 160 g·L^-1^ for women set by some sport federations, was never exceeded by any individual at any time, despite infusion of one and two units of RBCs. Consequently, it will be challenging at the least, to use any of the measured variables to discriminate blood samples taken before and after blood manipulation with cryo-preserved blood. For one, in some Control individuals, variations in hematological variables over time surpasses the effects of RBC re-infusion in the Transfusion group.

Multivariate statistical methods (PCA, OPLS and OPLS-DA) were applied on hematological variables to separate the samples from clean Control subjects, subjects before and after both blood donation and RBC re-infusion (Fig 7). Models were created at all sampled time points up to four weeks after RBC re-infusion. No valid model for complete separation of groups based on any, or a combined score of all, included hematological variables could be reached. The highest cumulative explained fraction (R^2^) and prediction by cross validation (Q^2^) never reached 0.30 (Fig 8). The 48 h time point after RBC re-infusion can be considered the closest relevant sampling time in any athletic situation. Also, before this time, re-infused RBCs may not yet be released into circulation by the spleen [71, 72]. The model at 48 h after blood donation (Fig 9A) reached R^2^ = 0.76 and Q^2^ = 0.59 (significant by R1). Variables included are ranked according to importance in 9B, with EPC, [Hb] and Ret. % being the most important. Removing any variable will reduce both R^2^ and Q^2^. In panel 9C, the model is used to classify the prediction set to the nearest class, which resulted in one Transfused subject (H) incorrectly classified as Control. Using data from 48 h post RBC re-infusion (Fig 10A), the model reached R^2^ = 0.40 and Q^2^ = 0.01 (significant by R1), and the ranked variables listed in 10B. The model after RBC re-infusion (equivalent to doped athletes) predicted one Control as Transfused, in other words one false positive (Fig 10C). The predictions at each time point sampled (termed misclassification) is listed in Table 7, where in total 14 of 56 Clean samples (25%) were false positive.

The reason for the lack of predictive power in any and all hematological variables analyzed is to be found in Figs 3–7, where overlapping values makes complete separation of groups impossible, even when reducing dimensions and combining scores by PCA/OPLS/OPLS-DA.

In conclusion, re-infusion of one or two units cryopreserved RBC in both men resulted in significant improvements in both VO_2max_ and performance, and improvements were not strictly related to changes in any measured blood variables. Analyzed hematological variables before and after blood transfusion of cryo-preserved blood in both men and women could not be used to separate groups by applied multivariate statistical methods. In addition, some common limits used for detection of blood doping were rarely exceeded. Hematological variables as measured in the present study appear inadequate for detection of autologous blood doping. Hematological profiling by multivariate statistics could not reach the WADA stipulated (2016) false positive ratio of <0.001% for any valid blood doping test, at any time point investigated.

## Supporting Information

S1 File Physical performance from Part A of the Study. (XLSX)

S2 File Subject characteristics and Hematological variables from Part A of the Study. (XLSX)

S3 File Physical performance from Part B of the Study. (XLSX)

S4 File Hematological variables from Part B of the Study. (XLSX)
